# Cortical Glutamate and GABA Changes During Early Abstinence in Alcohol Dependence and Their Associations With Benzodiazepine Medication

**DOI:** 10.3389/fpsyt.2021.656468

**Published:** 2021-07-05

**Authors:** Guoying Wang, Wolfgang Weber-Fahr, Ulrich Frischknecht, Derik Hermann, Falk Kiefer, Gabriele Ende, Markus Sack

**Affiliations:** ^1^Department of Neuroimaging, Central Institute of Mental Health, Mannheim Medical Faculty, University of Heidelberg, Mannheim, Germany; ^2^Department of Addiction Medicine and Addictive Behavior, Central Institute of Mental Health, Mannheim Medical Faculty, University of Heidelberg, Mannheim, Germany; ^3^German Institute of Addiction and Prevention Research, Catholic University of Applied Sciences, Cologne, Germany; ^4^Therapieverbund Ludwigsmühle, Landau in der Pfalz, Germany

**Keywords:** alcohol dependence, glutamate, GABA, benzodiazepine, withdrawal, N-acetylaspartate, 1H-MR-spectroscopy

## Abstract

In this report, we present cross-sectional and longitudinal findings from single-voxel MEGA-PRESS MRS of GABA as well as Glu, and Glu + glutamine (Glx) concentrations in the ACC of treatment-seeking alcohol-dependent patients (ADPs) during detoxification (first 2 weeks of abstinence). The focus of this study was to examine whether the amount of benzodiazepine administered to treat withdrawal symptoms was associated with longitudinal changes in Glu, Glx, and GABA. The tNAA levels served as an internal quality reference; in agreement with the vast majority of previous reports, these levels were initially decreased and normalized during the course of abstinence in ADPs. Our results on Glu and Glx support hyperglutamatergic functioning during alcohol withdrawal, by showing higher ACC Glu and Glx levels on the first day of detoxification in ADPs. Withdrawal severity is reflected in cumulative benzodiazepine requirements throughout the withdrawal period. The importance of withdrawal severity for the study of GABA and Glu changes in early abstinence is emphasized by the benzodiazepine-dependent Glu, Glx, and GABA changes observed during the course of abstinence.

## Introduction

Although benzodiazepines (BZD) are often used in clinical management of alcohol withdrawal, little is known about their effects on cortical concentrations of gamma aminobutyric acid (GABA) and glutamate (Glu) in alcohol dependent patients (ADPs). The present study aimed to detect longitudinal changes of the Glu and GABA concentrations in the anterior cingulate cortex (ACC) during alcohol detoxification treatment, by using proton magnetic resonance spectroscopy (MRS). Furthermore, we hypothesized a potential role of BZD in these longitudinal dynamic changes.

The alcohol withdrawal syndrome is characterized by excessive glutamatergic neurotransmission and reduced GABA functioning as well as a reduced number of GABA_A_ receptors which regulate the chloride channel, both indicated as key features of alcohol-dependent neuroplasticity (1–3). Our previous studies had focused on the excitatory neurotransmitter Glu and other brain metabolites [total N-acetylaspartate (tNAA), and total choline (tCh)] (4–6) during acute withdrawal and continued abstinence in non-medicated ADPs. The ACC was chosen as region of interest due to its important role in alcohol dependence and relapse. Our previous findings indicated that ACC Glu concentrations were elevated in non-medicated ADPs during acute withdrawal and normalized after 2 weeks of abstinence. In addition, a lower ACC NAA level due to chronic alcohol consumption and partial recovery during continued abstinence was observed repeatedly (7, 8).

GABA is the primary inhibitory neurotransmitter, and abnormalities in synaptic inhibition mediated by GABAergic neurons are associated with alcohol dependence (9). This GABAergic dysfunction leads to neuronal disinhibition, adding to the hyperarousal of hyperglutamatergic states which are thought to be the basis for alcohol withdrawal (10). Since cortical GABA concentrations are small and the peak is hidden under the creatine resonance without spectral editing, only a few MRS studies have reported on the effects of alcohol dependence on concentrations of GABA, showing non consistent results (10–12). Moreover, recent studies report that brain GABA levels in healthy subjects decreased after administration of BZD (13, 14). However, although BZD are the first-line pharmacological treatment of alcohol withdrawal symptoms by enhancing the activation of GABAergic neurons (2), their role in modulating brain GABA and Glu is still not clear.

This report presents cross-sectional and longitudinal findings of single voxel MEGA-PRESS MRS, focusing on absolute quantification of GABA, Glu, and Glu + glutamine (Glx) concentrations in the ACC of treatment-seeking ADPs during detoxification and early abstinence. Our primary focus of this study was to explore, whether the amount of BZD administered to ameliorate withdrawal symptoms was associated with the extent of longitudinal changes of Glu, Glx, and GABA.

## Methods

The present study comprised 20 ADPs and 22 age- and sex-matched healthy controls (HCs). Patients seeking voluntary treatment for their alcohol use disorders were recruited from a specialized inpatient treatment facility. All participants provided written informed consent before study participation. The study was approved by the Ethics committee of the Medical Faculty Mannheim, Heidelberg University (2007-234N-M).

Twenty (15 males, 5 females) ADPs were scanned twice with MRS at 3 T (Siemens, TimTrio systems), during alcohol withdrawal (TP1: day 1 of detoxification) and on day 14 of abstinence (TP2), respectively. HCs (18 males, 4 females) were scanned once. Of the 20 ADPs, 15 patients received BZD medication after the 1st MRS measurement. BZD doses varied according to withdrawal severity, assessed via Clinical Institute Withdrawal Assessment for alcohol (CIWA-Ar) until withdrawal symptoms had subsided (see Table 1). CIWA-Ar scores reflect the number and intensity of typical alcohol withdrawal symptoms (e.g., sweating, tremor, headache, nausea) assessed by trained personal. Scores above 10 typically indicate the need for BZD treatment to prevent seizures or delirium tremens. Diazepam was the only BZD used in the present sample.

**Table 1:** Characteristics and ACC metabolites concentrations of the ADP and HC samples.

	**ADP (Mean ± SD)**	**HC (Mean ± SD)**	**F or *t***	***p***	**Partial Eta Squared**	**Observed power**
Age	45.70 ± 9.62	46.41 ± 11.67	0.214	0.345		
Male/Female	15/5	18/4	−0.27	0.789		
LDH_Total (g)	654,963 ± 301,312	55,481 ± 77,205	66.861	0.000	0.663	1.000
LDH_last 1 2Months (g)	66,842 ± 46,482	1,963 ± 2,117	16.529	0.000	0.327	0.977
Diazepam (mg)	59.00 ± 39.29	–				
CIWA_unmed (max)^#^	8.94 ± 4.63	–				
Glu TP1 (i.u.)	10.20 ± 1.14	9.56 ± 0.67	5.002	0.031	0.111	0.588
Glx TP1 (i.u.)	12.53 ± 1.19	11.86 ± 0.94	4.181	0.047	0.095	0.514
GABA TP1 (i.u.)	2.71± 0.77	2.70 ± 0.58	0.002	0.965	0.000	0.050
tNAA TP1 (i.u.)	13.41 ± 0.75	14.07 ± 0.64	9.261	0.004	0.188	0.844
tCr TP1 (i.u.)	12.23 ± 1.21	11.91 ± 1.14	0.805	0.375	0.020	0.141
Glu TP2 (i.u.)	9.80 ± 0.63					
Glx TP2 (i.u.)	12.31 ± 0.84					
GABA TP2 (i.u.)	2.55 ± 0.49					
tNAA TP2 (i.u.)	17.09 ± 0.91					
tCr TP2 (i.u.)	12.20 ± 0.82					

*ADP, alcohol dependent Patients; HC, healthy controls; BDZs, benzodiazepine; HC, healthy controls; Mean ± SD, Mean ± Standard Deviation; Glu, glutamate; Glx, glutamate plus glutamine; GABA, gamma aminobutyric acid; tNAA, total N-acetylaspartate;LDH, Lifetime drinking history; CIWA, clinical institute withdrawal assessment for alcohol*.

# *CIWA_unmed (max): maximum CIWA score of non-medicated patients before BZDs were administered*.

*TP1 (day 1 of detoxification before BZDs were administered); TP2 (day 14 of abstinence)*.

MRS data was obtained using a MEGA-PRESS sequence (15) with TR/TE = 3,000/68 ms, 192 averages (96 “edit on,” 96 “edit off”), voxel size 20 × 30 × 40 mm3 as we had previously used in other studies (16). The MEGA-PRESS employed was based on the Siemens WIP package for VB15 which was ported to VB17 and expanded to include control of the reflection frequency. The editing pulse (gauss shape, duration: 20.36 ms, bandwidth: 44 Hz) was mirrored at 1.7 ppm, thus suppressing MM contributions in the GABA signal (17). Due to the low sensitivity of the GABA signal we chose a bigger voxel – suitable for GABA detection compared to our previous Glu study (4). For absolute quantification, an additional water-unsuppressed PRESS spectrum was acquired with TR/TE = 10,000/30 ms to minimize water relaxation effects. No other measurements except localizer and a 3D-anatomical MPRAGE were conducted preceding MEGA-PRESS spectroscopy.

The acquired raw data (Siemens' “twix” files) was post-processed via an in-house-developed algorithm written in MATLAB (The Mathworks Inc.). After reading the raw data, the single coil elements were phased and weighted. Weighting was based on coil SNR. The coil elements were then combined and the water-unsuppressed data was not further processed. The individual averages of the water-suppressed data were split in “edit on” and “edit off” and further processed separately but in the same manner. Correction of spectral misalignments was done by adjusting frequency and phase using a Nelder-Mead simplex algorithm (implemented in MATLAB's “fminsearch”) minimizing the least square error between the single spectra and a template within a predefined area in the frequency domain. As template the very first obtained “edit on” and “edit off” spectra were chosen, respectively, and a frequency range of 2.1–5.8 ppm for alignment correction was selected, thus covering the water and metabolites signal except NAA. Furthermore, to minimize the influence of noise on the adjustment, the spectra underwent temporarily an apodization of 15Hz during correction. To identify outlier spectra, the absolute sum of square differences of all spectra to their median spectrum in a range of 1.85–4.2 ppm was calculated. Averages were excluded in case of a more than three scaled median absolute deviation (MATLAB's “isoutlier”). This procedure of finding outliers and redetermination of the median was repeatedly done until no more outliers were detected. Exclusion of spectra was always pairwise. Lastly, the two resulting “edit on” and “edit off” spectra were aligned to each other, subtracted and saved for further quantification.

**Figure 1:**
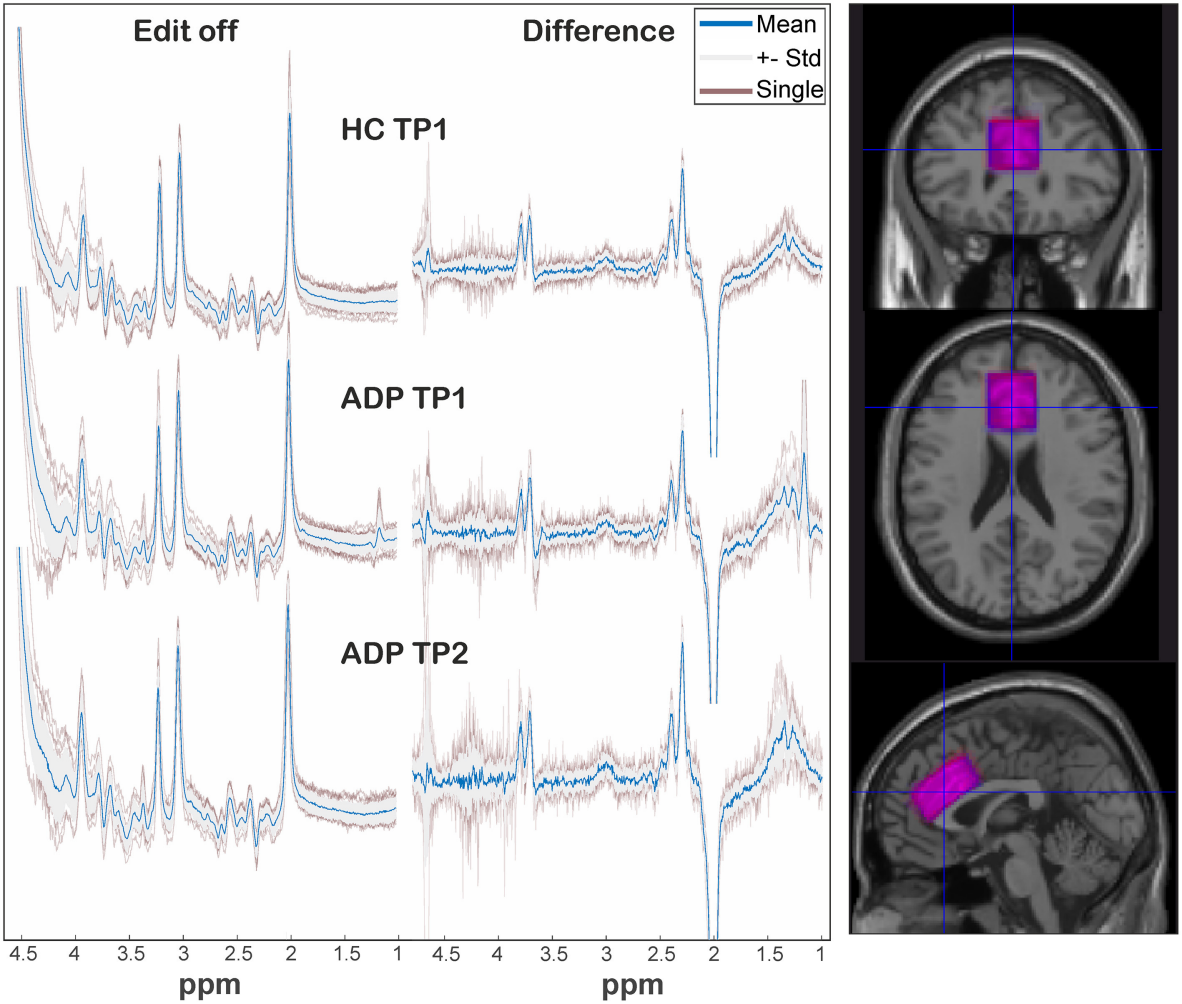
On the left: representation of spectra among groups and sessions. The individual spectra (transparent red) are overlaid by the standard deviation (gray area) and corresponding mean spectrum (blue). On the right: overlap of mean voxel location of HC (blue) and ADP (red) at TP1 after transformation in a template space.

Quantification was performed with LCModel (v6.3-1k) using a basis dataset which was created with VeSPA (https://scion.duhs.duke.edu/vespa/project) and the available “MEGA-PRESS” pulse sequence simulation. It includes the resonances of the following metabolites: GABA, Gln, Glu, GSH, NAA, Cr, NAAG, Eth, mI, Lac, Ala, Asp, Tau, Scyllo, GPC, PCr, PCh, Glyc (for difference spectrum: GABA, Gln, Glu, GSH, NAA, NAAG, Eth, Glyc). As recommended the option “SPTYPE = ”mega-press-3”” was used for the difference spectra. Absolute quantification was done based on (18) with voxel tissue compartments calculated by “SegSpec” (19) and, thus, adjusted for chemical shift displacement. Water and metabolite relaxation values were taken from (12) and (20–22), respectively, whereby the latter were averaged for GM and WM. All spectra underwent visual inspection by MRS experts to rate spectral quality and identify any spectra of poor quality. Furthermore, all LCModel Cramér Rao Lower Bounds of the analyzed metabolite signals were <20%. The voxel location as well as spectra are shown in Figure 1. The GABA levels were obtained from the “difference” spectra, all other reported metabolite levels from the “edit-off” spectra.

### Statistics

Cross-sectional analysis of the absolute quantified metabolite levels were performed using multivariate analysis of variance (MANOVA) with group as between-subject factor and four dependent variables (GABA, Glu, Glx, tNAA). For longitudinal analysis repeated measurements ANOVA with total BZD dosage as covariate were used, followed by simple correlations between total BZD dosage and metabolites' respective difference values. We regarded *p*-values < 0.05 as significant. All second level analyses were preformed using SPSS (IBM SPSS Statistics 26).

To investigate the comparability of spectral quality and voxel location among groups and sessions, frequency drifts, linewidths evaluated by LCModel and voxel overlap were analyzed.

Although the hypothesis is clearly directed to Glu, we decided to present Glu and Glx since the MEGA-PRESS sequence is not optimal to distinguish both measures.

## Results

### Cross-Sectional Group Comparisons on Day 1 of Detoxification (TP1)

On day 1 of detoxification, a MANOVA analysis yielded a significant lower ACC tNAA concentration [F (1, 40) = 9.261, *p* = 0.004, partial η^2^ = 0.188), higher ACC Glu [F (1,40) = 5.002, *p* = 0.031, partial η^2^ = 0.111] and higher Glx levels [F (1,40) = 4.181, *p* = 0.047, partial η^2^ = 0.095] in ADPs compared to HCs. Neither tCr, nor GABA concentrations differed significantly between groups (all *p* > 0.1) (see Table 1).

### Longitudinal Changes in ADPs of Glu, Glx and GABA Are Associated With BZD Dosage

Since we aimed to investigate the influence of BZD on the neurotransmitters Glu and GABA during detoxification and continued abstinence, we evaluated the correlation between the amount of BZD received for amelioration of withdrawal symptoms and Glu, Glx, and GABA levels. For this we used the subgroup of patients that received BZD (*n* = 15, 11 male, 4 female, age = 47.07 ± 8.34). Additionally, we used the amount of BZD as a covariate in a repeated measurements ANOVA analysis.

**Figure 2:**
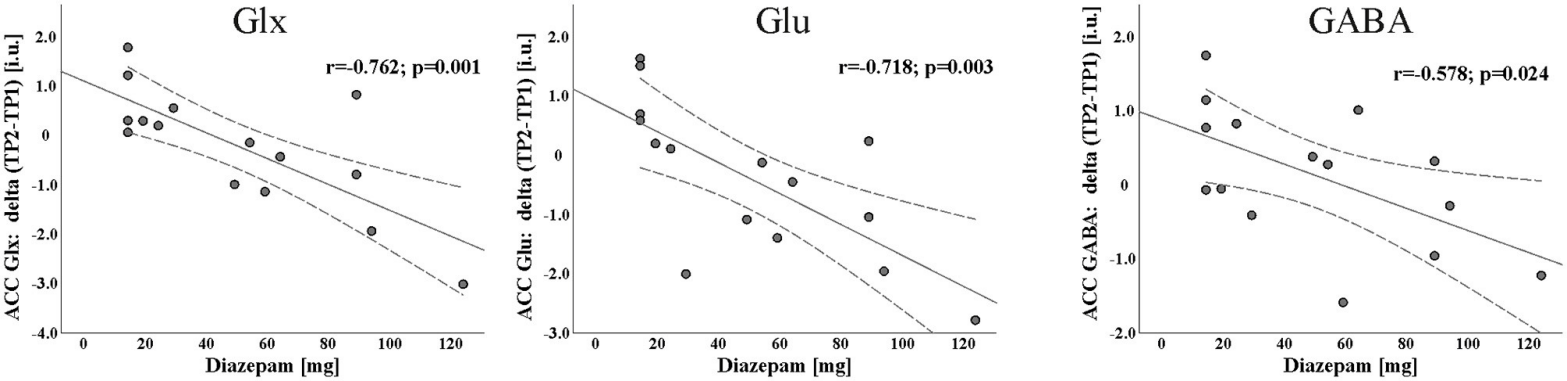
Scatterplot with linear fit line of the significant negative association between Diazepam and longitudinal changes of Glx, Glu, and GABA concentrations during first 2 weeks of abstinence [day14 of abstinence (TP2) - day 1 of detoxification (TP1)].

The amount of BZD received was negatively correlated with the change of Glx (*r* = −0.762, *p* = 0.001), Glu (*r* = −0.718, *p* = 0.003) and GABA (*r* = −0.578, *p* = 0.024) between TP1 and TP2 (TP2-TP1). No such relation was observed for tCr (*r* = −0.345, *p* = 0.208) and tNAA (*r* = −0.128, *p* = 0.650) although tNAA levels increased significantly [F(1, 13) = 9.563, *p* = 0.009, partial η^2^ = 0.424] in ADPs from TP1 to TP2 (see Figure 2).

**Figure 3:**
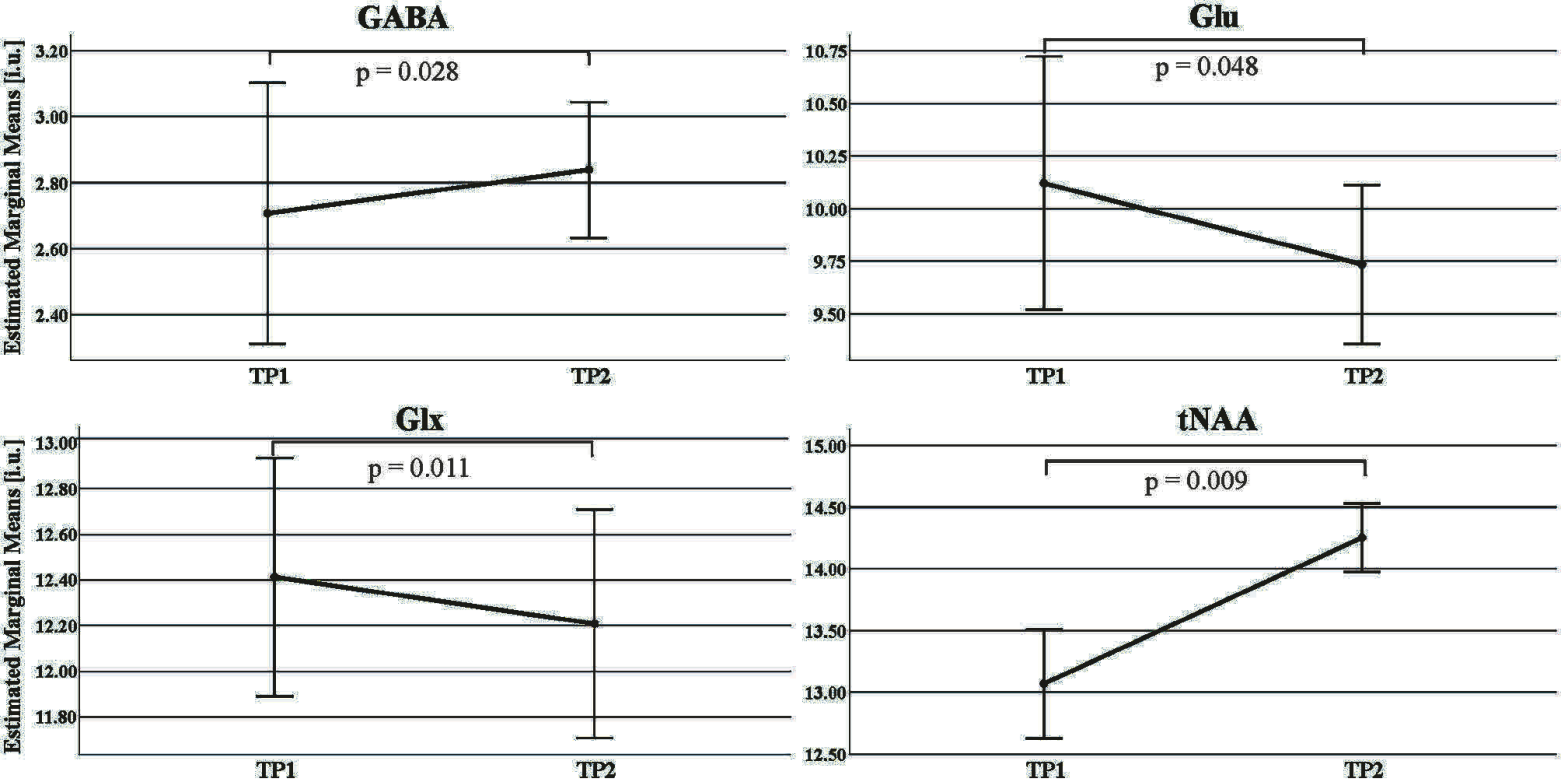
Estimated Marginal Means and standard error of GABA, Glu, Glx, tNAA concentrations in the ACC in ADP across two MRS scans: TP1 (day 1 of detoxification); TP2 (day 14 of abstinence). Estimated marginal means: mean value adjusted for covariate (amount of Diazepam) in the repeated ANOVA model.

We found a significant increase in GABA levels [F (1, 13) = 6.147, *p* = 0.028, partial η^2^ = 0.321, TP1: 2.71 ± 0.82 i.u., TP2: 2.84 ± 0.36 i.u.] as well as significant decreases in Glx [F (1, 13) = 8.799, *p* = 0.011, partial η^2^ = 0.404, TP1: 12.41 ± 1.18 i.u., TP2: 12.20 ± 0.88 i.u.] and Glu levels [F (1, 13) = 4.758, *p* = 0.048, partial η^2^ = 0.268, TP1:10.12 ± 1.28 i.u., TP2: 9.74 ± 0.68 i.u.] after 2 weeks of abstinence in the 15 patients receiving BZD. BZD dosage showed a significant interaction effect with all three neurotransmitter measures (see Figure 3).

### Comparability of Spectroscopic Measurements Between Groups

Figure 1 depicts a visual overview of acquired spectra and voxel overlap of HCs and ADPs at TP1. Analysis of standard deviations of individual frequency drifts [HC_TP1_: 0.73 ± 0.33; ADP_TP1_: 0.86 ± 0.24; ADP_TP2_: 0.72 ± 0.29; mean ± SD (Hz)] and linewidths [HC_TP1_: 0.0367 ± 0.0037; ADP_TP1_: 0.038 ± 0.0046; ADP_TP2_: 0.0387 ± 0.0067; mean ± SD (ppm)] revealed no significant differences (all *p* > 0.1). The calculated individual voxel overlap in ADPs between TP1 and TP2 yielded a mean coverage of 90 ± 5 % (including one outlier with 78% coverage) and revealed no significant difference in bulk tissue fractions calculated as gray matter/(gray matter + white matter) (HC_TP1_: 0.594 ± 0.031, ADP_TP1_: 0.597 ± 0.046, ADP_TP2_: 0.602 ± 0.045; mean ± SD).

## Discussion

In line with our previous findings, we found decreased tNAA on day 1 of detoxification in ADPs compared to HCs and a significant increase in tNAA levels during the first 2 weeks of abstinence in ADPs. This was repeatedly and robustly found before (7), which speaks for the plausibility of the sample and the data. Moreover, although the present study consists of patients with and without severe withdrawal symptoms, indicated by big variations in the CIWA score and the BZD values, patients at the first day of abstinence showed higher Glu and Glx concentrations in the ACC. However, in compliance with our previous findings from a different sample (4, 23) our results support the glutamate hyperexcitability hypothesis of alcoholism (24), representing an up-regulated glutamatergic activity during alcohol withdrawal as a consequence of counterbalancing the effects of chronic alcohol intake.

The correlation results indicate patients with greater decline in ACC GABA levels after 2 weeks of abstinence have received higher amounts of Diazepam. This is in line with previous MRS findings, showing a decrease in GABA levels after BZD administration in healthy subjects (13, 14). Notably, contrasting with previous studies indicating that BZD have no effects on Glx and Glu levels in HCs (25–27), our finding suggests that with higher total BZD dosages the patients received, ACC Glx and Glu levels declined to a bigger extent between TP1 and TP2. Unlike other studies, which mostly investigate Glx (Glu) level changes after acute administration of BZD, our sample received prolonged administration of BZD.

Moreover, when including total BZD dosage as a covariate, the Glx and Glu levels showed significant decreases between day 1 of detoxification and after 14 days of abstinence. This finding is in line with (4) showing elevated Glu levels during acute alcohol withdrawal and their amelioration within 2 weeks of abstinence. Opposite to our findings, Umhau et al. (28) reported a non-significant trend for an increase of Glu in a placebo-treatment abstinent ADPs group. This discrepancy may be driven by different baseline levels of Glu between the two studies. Instead of a measurement during acute withdrawal before BZD were administered, their first scan was on day 5 of abstinence, when some ADPs already had 4 days of BZD treatment. Moreover, as they did not include a healthy control group in their study, no conclusions on relative Glu levels at the beginning of treatment can be drawn.

Like in the study of Mon A, et al. (10), the group comparisons on day 1 of detoxification did not yield significant differences for GABA levels in our study. But our longitudinal analyses that included BZD dosage as covariate showed comparable patterns of GABA increase as did another MRS study in ADPs (11). The finding of a decrease in GABA after alcohol injection in healthy participants supports a GABA decrease as a result of alcohol consumption rather than withdrawal from alcohol (29).

The MRS detectable metabolite concentrations of Glx, Glu and GABA largely represent the signal from the respective neurotransmitters stored in presynaptic vesicles (state-related intracellular levels) rather than representing transient synaptic activity. This background in mind, we assume that the inverse trajectories of Glu and GABA after 14 days of abstinence in ADPs are associated with withdrawal-induced imbalance and recovery from neuroadaptations in the glutamine/glutamate – GABA cycle (30) where glutamine acts as a precursor for the synthesis of Glu, which itself is the direct precursor of GABA.

Interactions between chronic alcohol exposure, receptor expressions and binding potentials are beyond the scope of this article and are not yet fully understood (31). In addition, glutamatergic and GABAergic system modulations after chronic ethanol exposure are suggested to last over 120 days or even the whole life (9, 32). We assume that after the BZD treatment cessation the “hyperexcitable withdrawal-like” neurochemical state can occur repeatedly in ADPs over the long-term abstinence. This might manifest in heightened Glu levels caused by exposure to alcohol-related cues (smell, pictures) or social stress. Further research is needed to elucidate this hypothesis.

A limitation of the used MM suppression scheme is its susceptibility to frequency drift. Although neither a significant difference between TP1 and TP2 in frequency drift nor its associations with spectral quality were found, numerical differences point to slightly more movement in ADPs at TP1. Therefore, as drift can induce both increases and decreases of the GABA signal (33), we cannot completely rule out that this affected our findings of increased GABA levels.

Despite the limitations of a small sample size and a voxel that is much bigger and contained more white matter compared to our previous study on Glu changes during alcohol withdrawal (4), our findings corroborate our own previous studies and those of other groups. Addressing the question raised by (11) our results furthermore indicate that withdrawal severity during the full period of withdrawal (reflected by cumulated BZD needs) has to be taken into account when investigating neurotransmitter changes in ADPs during early abstinence.

## Data Availability Statement

The raw data supporting the conclusions of this article will be made available by the authors, without undue reservation.

## Ethics Statement

This study was reviewed and approved by the Ethics Committee of the Mannheim Medical Faculty of the Heidelberg University, Germany. All patients/participants provided their written informed consent to participate in this study.

## Author Contributions

GW was involved in data acquisition, statistical analysis, and writing of the manuscript. MS was responsible for MRS data, post-processing, and writing the manuscript. UF was involved in the planning of the study, patient recruitment and characterization, and interpretation of the results. WW-F was involved in statistical analysis and writing of the manuscript. GE was involved in the planning of the study, responsible for the supervision of the study, and coedited the manuscript. FK and DH were involved in the planning of the study and recruitment of ADP patients. All authors contributed to the article and approved the submitted version.

## Conflict of Interest

The authors declare that the research was conducted in the absence of any commercial or financial relationships that could be construed as a potential conflict of interest.
